# Supplementary figures and images for: Genome-wide analysis of the strigolactone biosynthetic and signaling genes in grapevine and their response to salt and drought stresses
Source: PeerJ. 2022 Jun 10;10:e13551. doi: 10.7717/peerj.13551 (PMC9196262; doi:10.7717/peerj.13551)

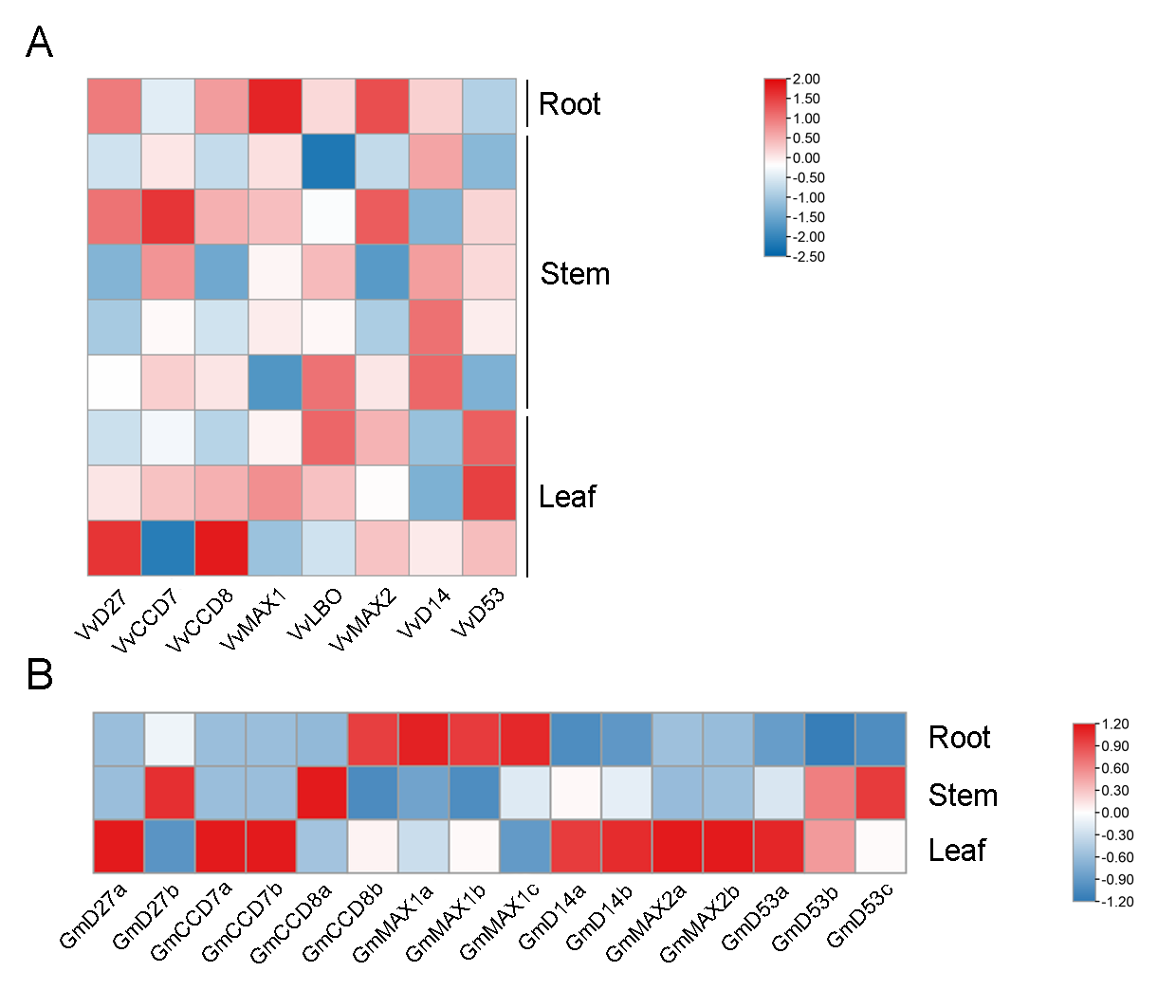

Supplement: Supplemental Information 1 — The heatmap of grapevine (A) and soybean (B) SL-related genes expression profiles in root, stem, and leaf tissues. [file peerj-10-13551-s001.png]

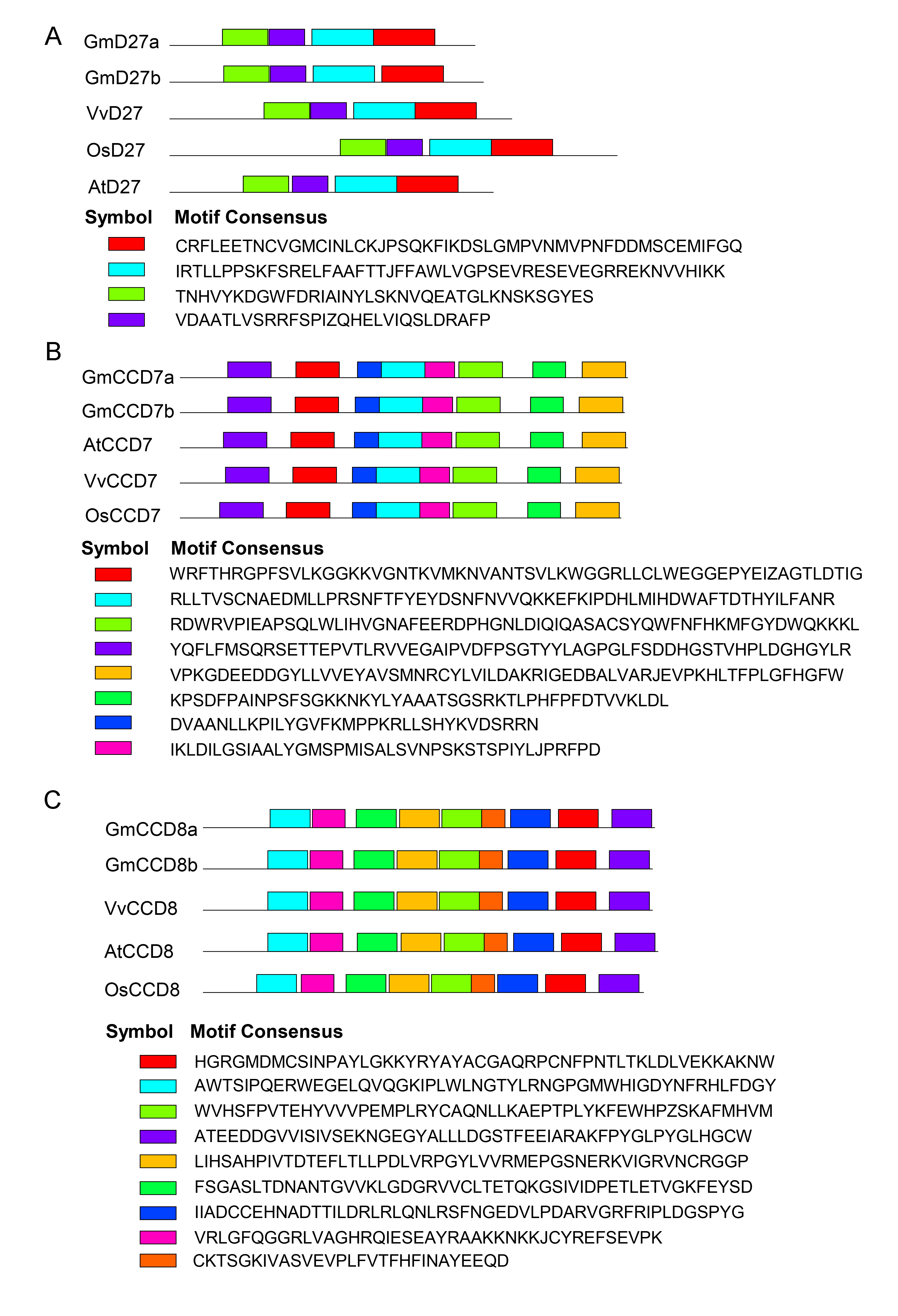

Supplement: Supplemental Information 2 — The motif composition of D27 (A), CCD7 (B), CCD8 (C) proteins was identified using the MEME online software, and the motifs were displayed by boxes of different colors. [file peerj-10-13551-s002.png]

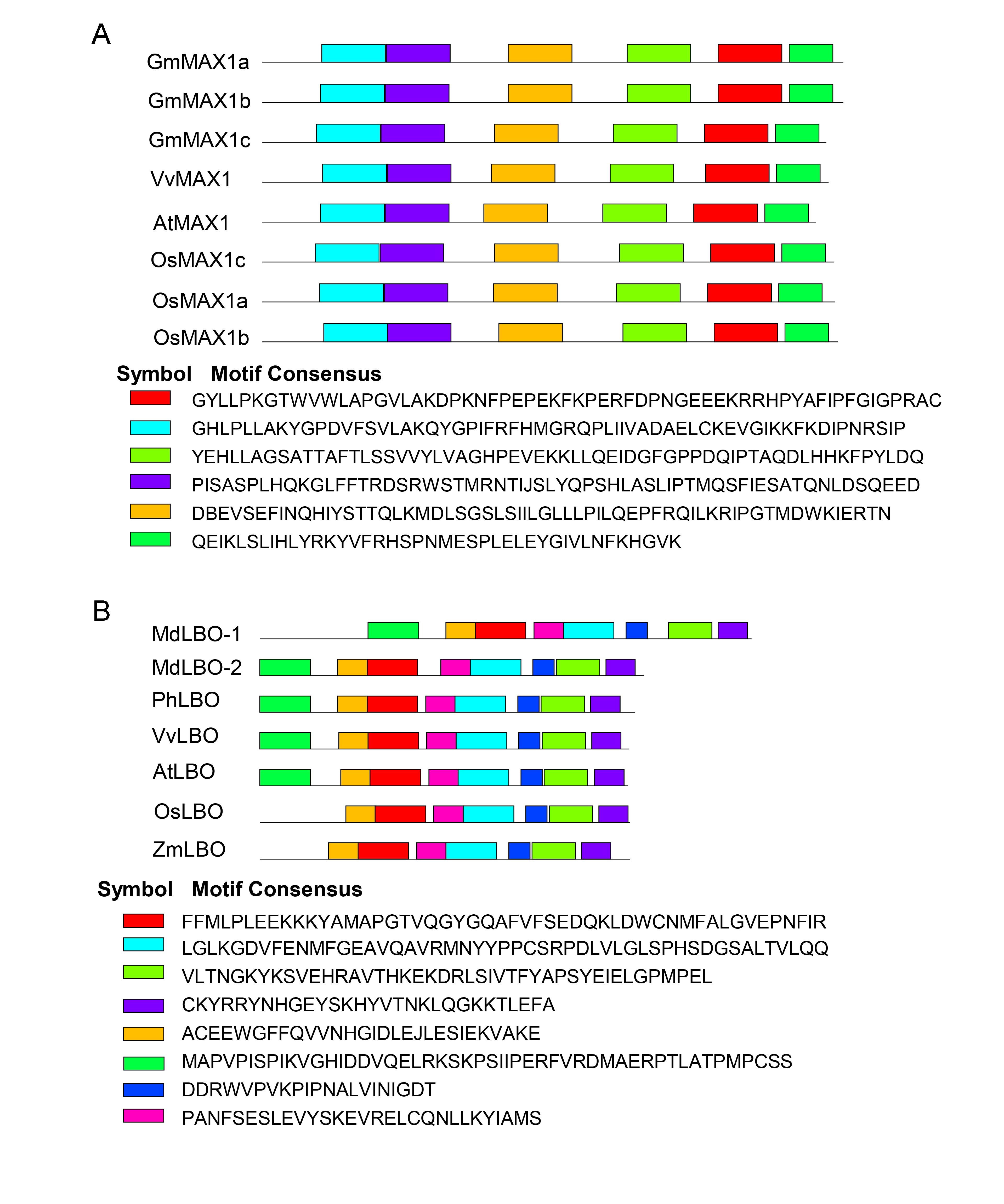

Supplement: Supplemental Information 3 — The motif composition of MAX1 (A), LBO (B) proteins was identified using the MEME online software. [file peerj-10-13551-s003.png]

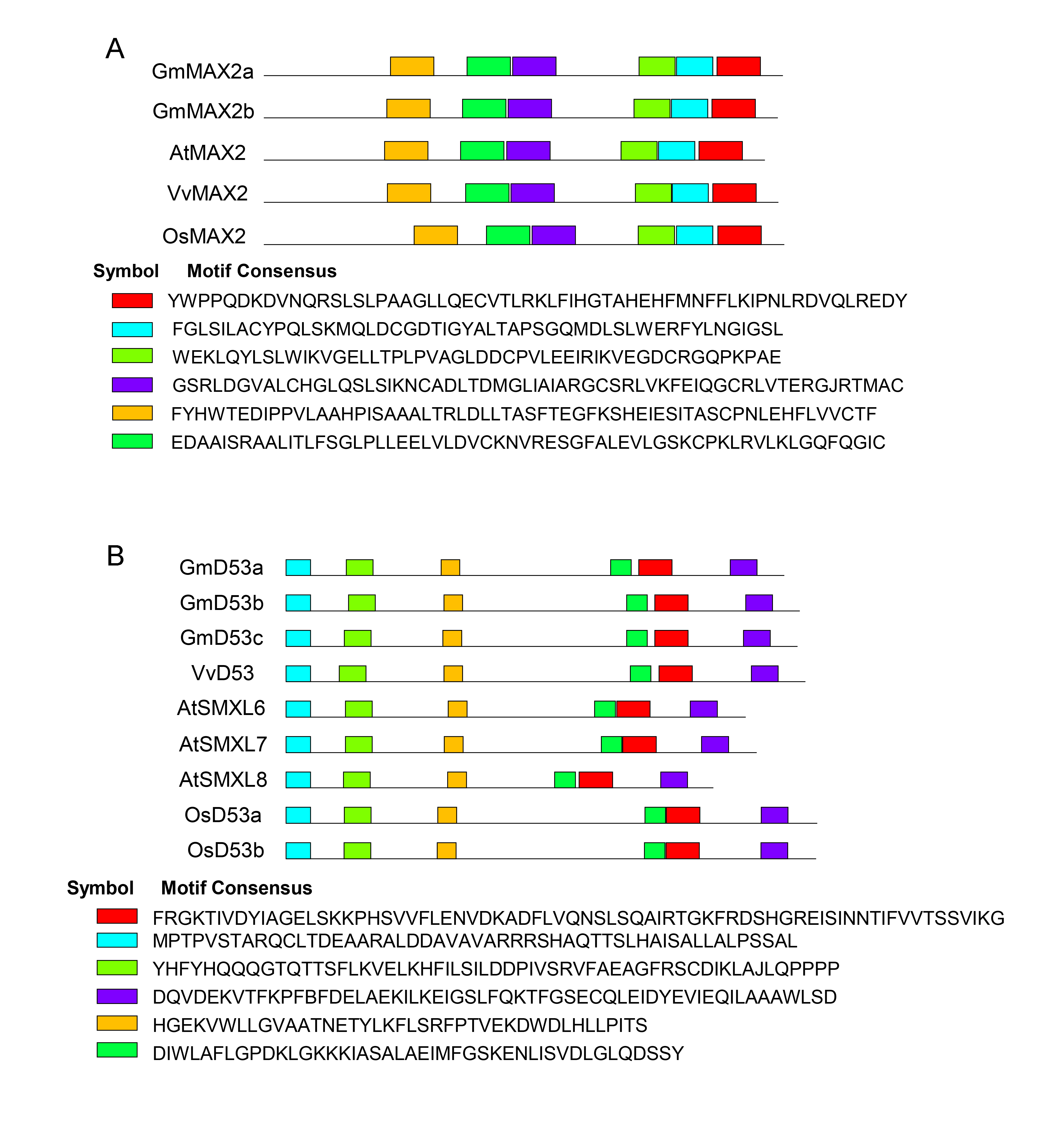

Supplement: Supplemental Information 4 — The motif composition of MAX2 (A), D53 (B) proteins was identified using the MEME online software. [file peerj-10-13551-s004.png]

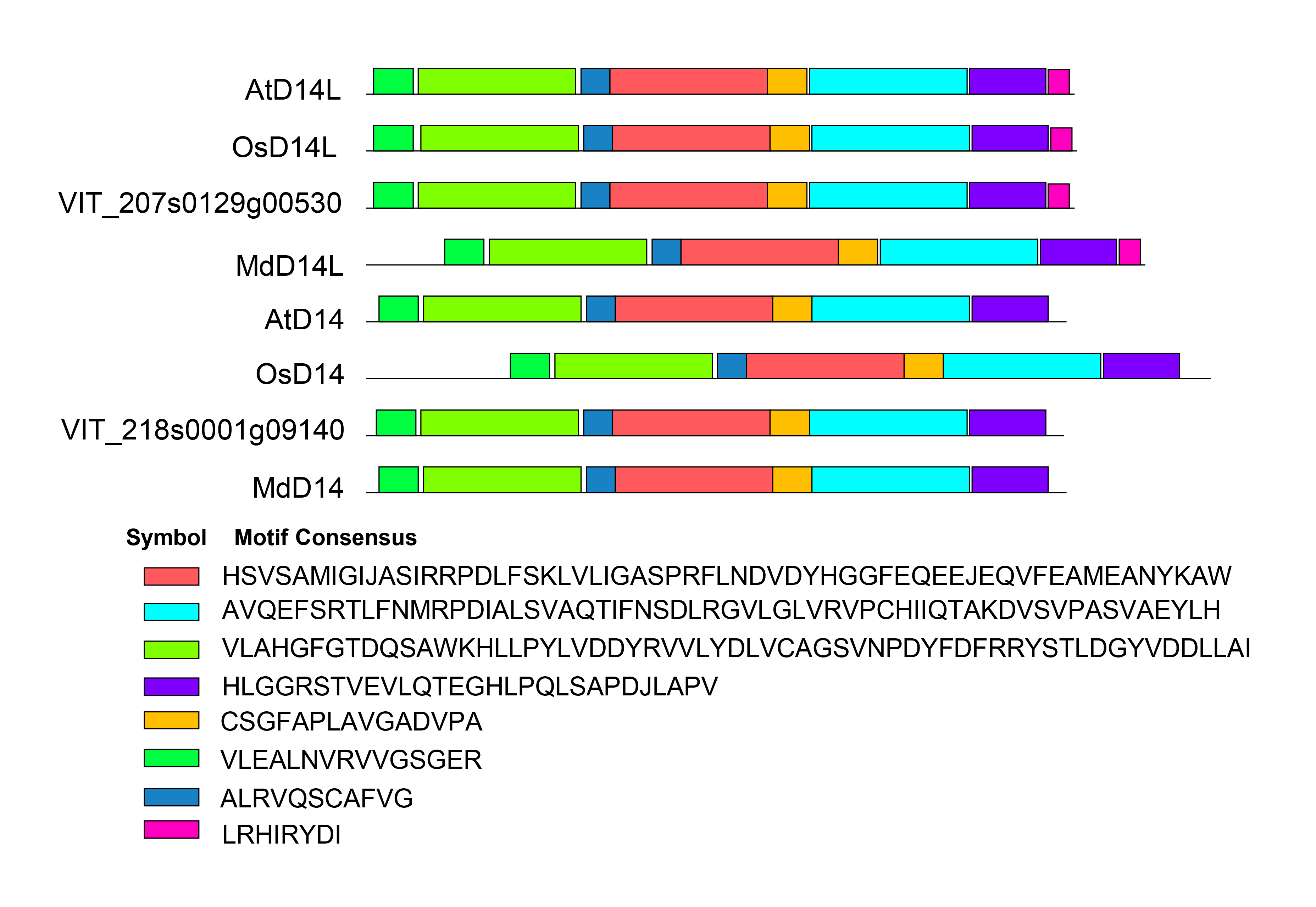

Supplement: Supplemental Information 5 — The motif composition of D14 proteins was identified using the MEME online software. [file peerj-10-13551-s005.png]
