# Supplementary material for: Genome-wide analysis of the strigolactone biosynthetic and signaling genes in grapevine and their response to salt and drought stresses
Source: PeerJ. 2022 Jun 10;10:e13551. doi: 10.7717/peerj.13551 (PMC9196262; doi:10.7717/peerj.13551)
Supplement: Supplemental Information 6 [file peerj-10-13551-s006.docx]

**Table S1 Gene-specific primers used in this study**

| Gene name | Primer sequence (5’-3’) |
| --- | --- |
| VvD27 F | Forward: TTTGCTGCCTTCACTACCTTGT |
| VvD27 R | Reverse: GGTTGAGACACATTCCCACAC |
| VvCCD7 F | Forward: TCCATCAATCCATCTTCCTCC |
| VvCCD7 R | Reverse: CCGTCGTCTTCTTCAATACCCT |
| VvCCD8 F | Forward: ATGAAGGTTCTGTGCCCTCG |
| VvCCD8 R | Reverse: TCTCCCGTCCAACAACAGTG |
| VvMAX1 F | Forward: CCGAGCATCCAGAAGTGGAG |
| VvMAX1 R | Reverse: TGCAAGAGACTTTGCCTGAT |
| VvMAX2 F | Forward: TCTCTTTGGATTGGCGTAGG |
| VvMAX2 R | Reverse: AAGTCCGTTTGCCCTGAAG |
| VvD14 F | Forward: TCTGAATGTCCGAGTAGTTGGG |
| VvD14 R | Reverse: CGACGAAGGCATCAAGAGTG |
| VvD53 F | Forward: TCCTTGCCTTTCCCTCATC |
| VvD53 R | Reverse: CTGATTCTGCTGCTGGAGGT |
| VvLBO-F | Forward: AGTCCATACCAGCAGCAATACC |
| VvLBO-R | Reverse: TCTCAAGCAAACCCAAGTCG |
| β-actinF | Forward: TCAGGAAGGACCTCTATGGC |
| β-actinR | Reverse: CTGTGGACAATGGATGGACC |

**Table S2 The function notes of *cis*-acting elements in the promoter sequences of grapevine SL-related genes**

| *Cis*-acting elements | Function of the *cis*-acting element |
| --- | --- |
| Phytohormone responsive | |
| P-box | gibberellin-responsive element |
| TATC-box | cis-acting element involved in gibberellin-responsiveness |
| TCA-element | cis-acting element involved in salicylic acid responsiveness |
| CGTCA-motif | cis-acting regulatory element involved in the MeJA-responsiveness |
| TGACG-motif | cis-acting regulatory element involved in the MeJA-responsiveness |
| ABRE | cis-acting element involved in the abscisic acid responsiveness |
| GARE-motif | cis-acting element involved in gibberellin-responsiveness |
| AuxRR-core | cis-acting regulatory element involved in auxin responsiveness |
| Plant development | |
| MBSI | MYB binding site involved in flavonoid biosynthetic genes regulation |
| MRE | MYB binding site involved in light responsiveness |
| HD-Zip 1 | element involved in differentiation of the palisade mesophyll cells |
| Box III | cis-acting regulatory element of protein binding site |
| CAT-box | cis-acting regulatory element related to meristem expression |
| circadian | cis-acting regulatory element involved in circadian control |
| Sp1 | cis-acting regulatory element involved in light responsive |
| GCN4_motif | cis-regulatory element involved in endosperm expression |
| O2-site | cis-acting regulatory element involved in zein metabolism regulation |
| GATA-motif | part of a light responsive element |
| I-box | part of a light responsive element |
| G-box | cis-acting regulatory element involved in light responsiveness |
| AE-box | part of a module for light response |
| Box 4 | part of a conserved DNA module involved in light responsiveness |
| TCT-motif | part of a light responsive element |
| L-box | part of a light responsive element |
| Abiotic stress responsive | |
| LTR | cis-acting element involved in low-temperature responsiveness |
| ARE | cis-acting regulatory element essential for the anaerobic induction |
| MYB | cis-acting element involved in MYB responsive and abiotic stress responsiveness |
| MYC | cis-acting element involved in MYC responsive and abiotic stress responsiveness |
| TC-rich repeats | cis-acting element involved in defense and stress responsiveness |
| GC-motif | enhancer-like element involved in anoxic specific inducibility |
| ERE | cis-acting element involved in the Ethylene responsiveness |

| **Gene name of SL-related gene in grapevine** | **Gene ID of SL-related gene in grapevine** | **Gene ID of SL-related gene in *Arabidopsis*** | **Gene name of SL-related gene in *Arabidopsis*** |
| --- | --- | --- | --- |
| D53 | VIT_206s0004g06700 | AT1G07200 | AtSMXL6 |
|  | VIT_206s0004g06700 | AT2G29970 | AtSMXL7 |
|  | VIT_206s0004g06700 | AT2G40130 | AtSMXL8 |
| MAX2 | VIT_212s0028g02140 | AT2G42620 | AtMAX2 |
| CCD8 | VIT_204s0008g03380 | AT4G32810 | AtCCD8 |
| CCD7 | VIT_215s0021g02190 | AT2G44990 | AtCCD7 |

**Table S3** **One-to-one orthologous relationships between grapevine and *Arabidopsis***

**Table S4** **One-to-one orthologous relationships between grapevine and rice**

| **Gene name of SL-related gene in grapevine** | **Gene ID of SL-related gene in grapevine** | **Gene ID of SL-related gene in rice** | **Gene name of SL-related gene in rice** |
| --- | --- | --- | --- |
| D53 | VIT_206s0004g06700 | Os11t0104300 | OsD53a |
|  | VIT_206s0004g06700 | Os12t0104300 | OsD53b |
| D14 | VIT_218s0001g09140 | Os03t0203200 | OsD14 |
| LBO | VIT_205s0020g01310 | Os01t0935400 | OsLBO |
| CCD7 | VIT_215s0021g02190 | Os04t0550600 | OsCCD7 |
